# Metabolomics for predicting hyperglycemia in pregnancy: a protocol for a systematic review and potential meta-analysis

**DOI:** 10.1186/s13643-019-1129-y

**Published:** 2019-08-24

**Authors:** Bianca Fioravanti Nicolosi, Debora F. Leite, Jussara Mayrink, Renato T. Souza, José Guilherme Cecatti, Iracema de Mattos Paranhos Calderon

**Affiliations:** 1 Department of Gynaecology and Obstetrics, Botucatu Medical School, São Paulo State University-UNESP, Botucatu, São Paulo Brazil; 2 Department of Obstetrics and Gynaecology, School of Medicine, University of Campinas (UNICAMP), Campinas, São Paulo Brazil

**Keywords:** Gestational diabetes mellitus, Hyperglycemia in pregnancy, High-risk pregnancy, Metabolomics, biomarkers

## Abstract

**Background:**

Hyperglycemia in pregnancy (HIP) has been recently differentiated between diabetes in pregnancy (DIP) and gestational diabetes mellitus (GDM). The proposed protocol is relevant, and clinical concern is due to the higher risk of adverse pregnancy outcomes (APO) and long-term effects on both the mother and the fetus. Fasting plasma glucose level (FPG) and oral glucose tolerance test (OGTT) are current diagnostic tools. However, controversy persists concerning diagnostic criteria, cut-off points, and even selective or universal screening. The objective of this systematic review is to assess the performance of metabolomic markers in the prediction of HIP.

**Methods:**

This is a protocol for a systematic review with potential meta-analysis. The primary outcome is GDM, defined as glucose intolerance identified in the second and third trimesters of pregnancy (any FPG ≥ 92 mg/dL and < 126 mg/dL OR when 75-g OGTT shows one altered value among these: FPG ≥ 92 mg/dL or 1-h post glucose load ≥ 180 mg/dL or 2-h post glucose load ≥ 153 mg/dL); the secondary outcome is HIP, defined as hyperglycemia detected in the first trimester of pregnancy (any FPG ≥ 126 mg/dL). A detailed systematic literature search will be carried out in electronic databases and conference abstracts, using the keywords “gestational diabetes mellitus,” “metabolomics,” “pregnancy,” and “screening” (and their variations). We will include original peer-reviewed articles published from Jan 1, 1999, to Dec 31, 2018. Original studies including diabetes diagnosed before pregnancy (T2DM and T1DM), multiple pregnancies, and congenital malformations will be excluded. All results regarding samples, participant characteristics, metabolomic techniques, and diagnostic accuracy measures will be retrieved and analyzed. Since this is a systematic review, no ethical approval is necessary.

**Discussion:**

This systematic review may have the potential to provide significant evidence-based findings on the prediction performance of metabolomics. There are short and long-term repercussions for the mother and the newborn. Therefore, both may benefit from an accurate prediction technique for HIP.

**Systematic review registration:**

This protocol was registered in the PROSPERO platform under number CRD42018100175.

**Electronic supplementary material:**

The online version of this article (10.1186/s13643-019-1129-y) contains supplementary material, which is available to authorized users.

## Background

The current classification of hyperglycemia in pregnancy (HIP) distinguishes diabetes mellitus in pregnancy (DIP), diagnosed before 20 weeks, from gestational diabetes mellitus (GDM), identified in the second or third trimester of gestation [1, 2]. When criteria for GDM are not fully met, less severe degrees of GDM also produce adverse effects on the mother and newborn [3–5]. Nevertheless, women with mild gestational hyperglycemia (MGH)—those with hyperglycemia in the glycemic profile, despite a normal response to oral glucose tolerance test (OGTT)—have been identified in specialized obstetrical centers [6, 7]. This subgroup of women is at higher risk of maternal and perinatal adverse outcomes, suggesting that their glycemic levels should also be controlled during pregnancy. More recently, despite several new criteria and cut-off points proposed for the diagnosis of GDM [1, 8], it is estimated that MGH women account for more than 15% of cases. Therefore, women with MGH are more likely to be identified and treated as those with GDM [9].

Universal screening for gestational diabetes is initially based on a fasting plasma glucose (FPG) measurement during the first prenatal visit (preferably before 20 weeks). DIP is diagnosed when FPG ≥ 126 mg/dL or GDM is considered when FPG is ≥ 92 mg/dL and < 126 mg/dL. An OGTT of 75 g is recommended between 24 and 28 weeks of gestation, when initial FPG < 92 mg/dL, and GDM is considered when FPG ≥ 92 mg/dL or 1-h post glucose load ≥ 180 mg/dL or 2-h post glucose load ≥ 153 mg/dL [1, 8]. This recommendation is still controversial due to the high costs involved and the lack of long-term evidence of any benefit [2, 10]. In this context, race/ethnicity, family history, body mass index (BMI), and prior history of GDM have been tested as the first screening tools for GDM in prediction models. However, their modest results may be due to low prevalence rates (reaching a maximum of 50% in pregnant women with GDM) and insufficient accuracy. This might lead to diagnostic testing in many women who would not require testing, particularly during late pregnancy [11–14].

The earlier the diagnosis and treatment, the better the prognosis of HIP. Thus, early identification of women at risk is critical. Research efforts have targeted first-trimester data collection. Many types of biomarkers have been tested, alone or in prediction models. Lower levels of adiponectin, sex-hormone-binding globulin, and high levels of C-reactive protein have been associated with an increased risk for GDM [15–17]. Likewise, studies have provided insight into the prediction of diabetes using metabolites of different chemical classes, resulting in diverse prediction performances. The advantage of metabolomics is that it has the potential to capture disease-relevant metabolic changes and identify novel biomarkers of disease processes [18].

Metabolomic studies in GDM have addressed biomarkers for early risk stratification, therapeutic effects of insulin and dietary treatment, and the risk of transition from GDM to short- or long-term T2DM. Several studies have identified some metabolites for GDM such as amino acids and their derivatives, organic acids, lipids, and fatty acids. However, they are extremely diverse in several aspects: (i) clinical controversies in GDM diagnostic protocol, (ii) gestational age for participant inclusion, (iii) biological samples (blood, urine) used, and (iv) instrumental platforms employed— mass spectrometry (MS), liquid chromatography (LC), gas chromatography (GC) or capillary electrophoresis (CE), or proton nuclear magnetic resonance (^1^H-NMR) spectroscopy [19–24]. These variables may explain the inconsistent findings [25].

This scenario and the lack of a systematic review (SR) on metabolomic markers as risk predictors to HIP (DIP or GDM) justify the proposition of this protocol. The source or type of the samples evaluated and the different analytical techniques will be evaluated; the results will help define the role of biomarkers in the identification of women at risk of hyperglycemia during pregnancy. Thus, the objective of the proposed SR is to assess the performance of metabolomic markers in pregnant women to predict HIP.

## Methods/design

### Review question

As a result of the social and economic implications of HIP disorders, their consequences to maternal and fetal lives worldwide, in addition to the increasing applicability of omics technologies, this systematic review will be guided by the following research question: “What is the performance of metabolomic markers in pregnant women to predict HIP, independently of DIP or GDM?”

### Condition or domain studied

Pregnant women with hyperglycemia in pregnancy (HIP)—categorized as gestational diabetes mellitus (GDM) or diabetes in pregnancy (DIP) [1, 2]—is the condition to be assessed.

### Participants/population

We will include original studies involving pregnant women with hyperglycemia, irrespective of the diagnostic criteria for this condition.

### Interventions/exposure

The intervention to be analyzed is screening for HIP (GDM or DIP) with the metabolomic approach. Maternal samples need to be drawn earlier than standardized recommendations for hyperglycemia screening in pregnancy.

### Study inclusion and exclusion criteria

Inclusion criteria: Original cohort or case-control studies including women who underwent any screening test for hyperglycemia or GDM during pregnancy.

Exclusion criteria: Studies on pregnant women with previous diabetes mellitus, either type 1 or type 2, diagnosed before metabolomic screening during pregnancy. In addition, all studies including pregnant women under health conditions that can affect, directly or indirectly, the levels of glycemia across pregnancy, that is, pancreas or thyroid cancer, severe obesity, chronic use of corticosteroids, and others, will also be excluded.

### Outcomes

The primary outcomes will be the diagnosis of GDM or hyperglycemia, regardless of the diagnostic criteria applied.

### Literature search

The primary source of information will be the electronic databases: PubMed, EMBASE, LILACS, SciELO, CINAHL, MIDIRS, Scopus, and Web of Science. Secondary sources will include Google Scholar, hand-searching the reference list for eligible studies, conference proceedings, and direct contact with authors, when necessary. In addition, the reference’s list of each selected article will be checked for finding some study not yet identified with the previous procedures.

The original search strategy proposed is the follows: “#1 “Metabolomics”[Mesh] OR Metabolomic OR Metabonomics OR Metabonomic; #2 “Diabetes, Gestational”[Mesh] OR Diabetes, Pregnancy-Induced OR Diabetes, Pregnancy Induced OR Pregnancy-Induced Diabetes OR Gestational Diabetes OR Diabetes Mellitus, Gestational OR Gestational Diabetes Mellitus; #3 “Pregnancy in Diabetics” [Mesh] OR Pregnancy in Diabetic OR Pregnancy in Diabetes OR Pregnancy in Diabete; #4 “Hyperglycemia”[Mesh] OR Hyperglycemias OR Hyperglycemia, Postprandial OR Hyperglycemias, Postprandial OR Postprandial Hyperglycemias OR Postprandial Hyperglycemia; #5 “Pregnancy”[Mesh] OR Pregnancies OR Gestation; #1 AND #2 OR #3 OR (#4 AND #5)”*.*

The keywords linked to the outcomes of interest (hyperglycemia OR gestational diabetes screening) will be combined with terms related to metabolomic (markers, samples, and technique) and pregnancy, using Boolean connectors and specific Mesh. The same search strategy will be applied for each database, adapting for individual filters, main language, their own syntax, and mechanisms of search. The complete search strategy is provided as Additional file 1.

Studies published in the last 20 years (from Jan 1, 1999, to Dec 31, 2018) will be taken into account. The search strategy will be re-run before final analysis, to check for recently published eligible studies. There will be no language restrictions; selected studies in a language different from English, French, Spanish, and Portuguese will be translated and considered for eligibility.

### Data extraction and management

Literature search, study selection, and data extraction will be performed independently by two investigators (BFN, DFBL). Firstly, titles and abstracts will be assessed, and when more data is needed, the full texts will be read to decide on study inclusion. All disagreements will be discussed until a consensus is reached, and when necessary, with the collaboration of a third senior investigator. Academic supervisors (IMPC, JGC) will resolve any discrepancies and will be responsible for checking and approving each step of the review.

Variables to be extracted include authors and year of publication, study design, definition or protocol used for GDM/Hyperglycemia diagnosis, number of participants enrolled (pregnant women screened with and without GDM/Hyperglycemia), gestational age of assessment (both for metabolomics and for standardized GDM diagnosis), laboratory methods applied (e.g., technique, year of sample collection, year of laboratory analysis), and biological sample analyzed (e.g., blood, amniotic fluid). Authors will be contacted in case any additional data is required. The Kappa index will be used to evaluate the degree of agreement in the selection and data extraction performed by two different investigators (BFN, DFBL).

### Risk of bias assessment

Both investigators initially involved with the literature search (BFN and DFBL) will assess the methodological quality of all included studies. They must check their criteria and discuss any discordance risk of bias. A third investigator should resolve any disagreement if necessary, and final decisions should be made by the majority. The “Quality Assessment of Diagnostic Accuracy Studies” (QUADAS-2) tool will be used [26]. This tool comprises 4 domains: patient selection, index test, reference standard, and flow and timing. Each domain is assessed in terms of risk of bias, and the first 3 domains are also assessed in terms of concerns regarding applicability. Signaling questions are included to help judge the risk of bias. The QUADAS-2 tool is applied in 4 phases: summarize the review question, tailor the tool and produce review-specific guidance, construct a flow diagram for the primary study, and judge bias and applicability. This tool will allow for a more transparent rating of bias and applicability of primary diagnostic accuracy studies.

### Strategy for data synthesis

An aggregate pregnant women data synthesis will be performed with all included studies. Narrative data will be analyzed and structured according to the results of diagnostic protocols applied. If possible, a meta-analysis (hierarchical summary of the receiver characteristic operating curve) of diagnostic accuracy measures will be conducted. For summarizing the effects in case a meta-analysis is feasible, a fixed effects model will preferably be employed if heterogeneity is judged to be low enough; otherwise, a random effects model will be used. If available data does not allow a quantitative synthesis, a qualitative descriptive approach will be used instead.

Regarding the Preferred Reporting Items for Systematic Reviews and Meta-Analysis (PRISMA) statement recommendations, a flow diagram will show details of the literature search and study selection [27]. Characteristics of the included studies and the risk of bias assessment will be shown in tables. Depending on data availability, we will perform subgroup analysis according to blood glucose levels (non-diabetic, hyperglycemia in pregnancy, and gestational diabetes mellitus).

### Potential limitations of this review

Metabolomics is a novel technology that does not have a unique and complete definition of methods, analytical platforms, and specimens to improve accuracy. Likewise, hyperglycemia in pregnancy has conflicting diagnostic criteria, cut-off points, and screening tests, in addition to an important gap in long-term maternal and fetal adverse outcomes. These issues, in conjunction with marked heterogeneity of the included population, may influence study quality, and thus the expected results.

### Ethics and dissemination

Since this is a systematic review protocol, no ethics committee approval is necessary. This protocol was registered in the International Prospective Register of Systematic Reviews (PROSPERO) database, number CRD42018100175, and adheres to the Preferred Reporting Items for Systematic Reviews and Meta-Analysis Protocols (PRISMA-P) statement [27].

Our research group is committed to the advance in the metabolomic field of reproductive health, performing systematic reviews (CRD42018089985; CRD42018097409; CRD42018100172) about metabolomic markers as predictive tools on adverse pregnancy outcomes. Therefore, this protocol will be electronically available at our website (http://www.medscinet.com/samba), and a report will be sent to our sponsors. The authors will submit the systematic review results for publication in peer-reviewed scientific journals.

### Patient and public involvement

There was no patient or public involvement in this systematic review protocol.

## Discussion

In general, GDM is only identified and treated at the end of the second trimester of pregnancy, after clinical diagnosis. Early identification of women at risk for GDM has been a major challenge because the ideal tool for this was not yet defined. Likewise, significant gaps still remain in the understanding of genetic and environmental risk factors, as well as related mechanisms that contribute to GDM. According to some authors, metabolome studies provide a general summary of metabolic interactions within a given biological system and allow the simultaneous identification and quantification of a large number of analytes in a highly productive and unbiased manner [28]. In this context, metabolomics emerged as a technology with a potential for the early detection of GDM, besides to favor the understanding of the pathogenesis and the impact of the disease in the mother and her offspring [29].

Our study intends to identify metabolomic markers that will potentially clarify some gaps in HIP (GDM or DIP), considering the different criteria for GDM/hyperglycemia in pregnancy and metabolomic techniques. Thus, a standardized and detailed protocol for a systematic review of early metabolomic predictors is of great importance to ensure significant evidence-based findings on the prediction performance of metabolomics. There are short- and long-term repercussions for the mother and the newborn, and both might benefit from an accurate prediction technique for HIP. Furthermore, the results of this systematic review may suggest new studies on the topic, focused on identifying the best technique for metabolomic screening.

## Additional file

Additional file 1: Metabolomics for predicting gestational diabetes mellitus: protocol for a systematic review and meta-analysis. (DOC 60 kb)

## Data Availability

This is a protocol for a systematic review, and therefore, no data are generated or analyzed. After completion, all search strategies, results, and compiled information will be included in the corresponding article to be published and stored in potential Supplementary Information files.

## Abbreviations

^1^H-NMR: Proton nuclear magnetic resonance spectroscopy
APO: Adverse pregnancy outcomes
BMI: Body mass index
CE: Capillary electrophoresis
DIP: Diabetes in pregnancy
FPG: Fasting plasma glucose levels
GC: Gas chromatography
GDM: Gestational diabetes mellitus
HIP: Hyperglycemia in pregnancy
LC: Liquid chromatography
MGH: Mild gestational hyperglycemia
MS: Mass spectrometry
OGTT: Oral glucose tolerance test
T1DM: Type 1 diabetes mellitus
T2DM: Type 2 diabetes mellitus
