# Supplementary material for: Metabolomics for predicting hyperglycemia in pregnancy: a protocol for a systematic review and potential meta-analysis
Source: Syst Rev. 2019 Aug 24;8:218. doi: 10.1186/s13643-019-1129-y (PMC6708156; doi:10.1186/s13643-019-1129-y)
Supplement: Supplementary file 1 — Metabolomics for predicting gestational diabetes mellitus: protocol for a systematic review and meta-analysis. (DOC 60 kb) [file 13643_2019_1129_MOESM1_ESM.doc]

**Metabolomics for predicting gestational diabetes mellitus: protocol for a systematic review and meta-analysis**

|  | # Date |  |
| --- | --- | --- |
| 1 | Gestational diabetes |  |
| 2 | Gestational diabetes mellitus |  |
| 3 | diabetes |  |
| 4 | Diabetes mellitus |  |
| 5 | GDM |  |
| 6 | hyperglycemia |  |
| 7 | MHL |  |
| 8 | Mild gestational hyperglyvemia |  |
| 9 | Mild gestational hyperglycemia |  |
| 10 | Hyperglicemia |  |
| 11 | Insulin resistance |  |
| 12 | Overt diabetes |  |
| 13 | Overt gestational diabetes |  |
| 9 | #1 OR #2 OR #3 OR #4 OR #5 |  |
| 10 | metabolomic* |  |
| 11 | metabonomic* |  |
| 12 | metabolit* |  |
| 13 | H NMR |  |
| 14 | proton NMR |  |
| 15 | proton nuclear magnetic resonance |  |
| 16 | liquid chromatogra* |  |
| 17 | gas chromatogra* |  |
| 18 | UPLC |  |
| 19 | ultra-performance liquid chromatograph* |  |
| 20 | ultra performance liquid chromatograph* |  |
|  | Metabol* profil* |  |
| 21 | #7 OR #8 OR #9 OR #10 OR #11 OR #12 OR #13 OR #14 OR #15 OR #16 OR #17 |  |
| 22 | pregnan* |  |
| 23 | antenat* |  |
| 24 | ante nat* |  |
| 25 | prenat* |  |
| 26 | pre nat* |  |
|  |  |  |
| 32 | Glucose concentrations |  |
| 33 | Fasting plasma glucose |  |
| 34 | GTT |  |
| 35 | OGTT |  |
| 36 | Glucose tolerance test* |  |
| 37 | Oral glucose tolerance test* |  |
| 39 | Glycemic curv* |  |
| 40 | Glycemic profil* |  |
| 41 | Hba1c |  |
|  | Glycosylated haemoglobin |  |
